# The Effect of Community-Based Education for Lifestyle Intervention on The Prevalence of Metabolic Syndrome and Its Components: Tehran Lipid and Glucose Study

**DOI:** 10.5812/ijem.5443

**Published:** 2013-07-01

**Authors:** Fereidoun Azizi, Parvin Mirmiran, Amir Abbas Momenan, Farzad Hadaegh, Ali Habibi Moeini, Firoozeh Hosseini, Saleh Zahediasl, Arash Ghanbarian, Farhad Hosseinpanah, and Members of Tehran Lipid and Glucose Study Group

**Affiliations:** 1 Endocrine, Obesity and Prevention of Metabolic Disease Research Center, Research Institute for Endocrine Sciences, Shahid Beheshti University of Medical Sciences, Tehran, IR Iran

**Keywords:** Metabolic Syndrome, Lifestyle, Community-Based, Prevalence

## Abstract

**Background:**

It has been shown that life style modification may decrease the prevalence of metabolic syndrome, but this intervention has not been reported in community setting.

**Objectives:**

Effect of lifestyle modification on prevalence of metabolic syndrome and its components were assessed in an urban population.

**Materials and Methods:**

In 6870 participants of Tehran Lipid and Glucose Study aged 20-74 years, the prevalence of metabolic syndrome and its components were measured before and after a 3.6 years interval. Lifestyle intervention was employed at a community level including 2961 individuals and also 3909 subjects which were recruited as controls. Logistic regression analysis was adjusted for age, sex and medications.

**Results:**

After 3.6 years, the rise in the prevalence of metabolic syndrome was less prominent in intervention than control group (*P* < 0.002 for increase of metabolic syndrome prevalence between groups), with an OR of 0.84 (confidence interval 0.75-0.95). After intervention the prevalence of abdominal obesity, elevated fasting glucose levels, elevated triglyceride and low HDL cholesterol were more prominent in control group, as compared to intervention group.

**Conclusions:**

Community based lifestyle modifications in Tehranian adults delayed rise in the prevalence of metabolic syndrome and some of its components.

## 1. Background

The metabolic syndrome is a clustering of non-communicable diseases (NCD) which may increase probability of developing such disorders like cardiovascular diseases, diabetes mellitus and kidney disease (1, 2). Risk of cardiovascular diseases accompanying metabolic syndrome is approximately doubled compared with absence of the syndrome (3); Moreover individuals with metabolic syndrome are at increased mortality risk of cardiovascular diseases and all other causes (2-4).

Studies have shown that lifestyle intervention reduces the risk of progression from glucose intolerance (IGT) to overt type 2 diabetes (5, 6). Furthermore, it has been shown that lifestyle modification may also decrease the prevalence of metabolic syndrome and reduce the occurrence of abdominal obesity in subjects with IGT (7). Such an effect in reducing prevalence of metabolic syndrome has been reported in a few studies in special groups (8). However, the evidence is limited regarding specific strategies that are most helpful for the long-term maintenance of lifestyle changes (9). In addition, implementation of a healthful lifestyle must expand beyond the individuals to the community scale and basically in cultural levels. Searching current literature shows that some nationwide health screening and interventions programs specifically targeting the metabolic syndrome has been designed (10), Despite the fact, the effect of lifestyle modification on management of metabolic syndrome has not been reported at the community scale yet.

The Tehran Lipid and Glucose Study (TLGS) provides a unique opportunity to begin to address these issues. It is a large scale community based prospective study performed on a representative sample of district 13 of Tehran inhabitants, the capital city of Iran. The TLGS was established with the aim of determining the prevalence of metabolic syndrome and evaluating the feasibility and effectiveness of lifestyle modification interventions in prevailing or postponing the development of the syndrome risk factors and outcomes in a population in nutrition transition (11). The baseline report of TLGS showed high prevalence of metabolic syndrome in the study population (12).

## 2. Objectives

The aim of this study was to assess the effects of lifestyle modifications on metabolic syndrome and some of its components in urban population of TLGS.

## 3. Materials and Methods

### 3.1. Design and Subjects

The rational and design of TLGS has been published previously in other studies (11, 13). It aims at determining the risk factors of cardiovascular diseases in a population of urban families in metropolitan Tehran in order to design effective strategies for controlling the incidence of risk factors and consequently their outcomes. The project was approved by the ethics committee of the National Research Council of Islamic Republic of Iran. Signed written informed consent has been obtained from each participant.

The TLGS design consisted of two stages; phase 1 was a cross-sectional prevalence study of NCD and its associated risk factors implemented from March 1999 to December 2001. Phase II was a prospective follow-up study which begun from January 2002. Data recollection was performed after a 3 year interval for all participants.

### 3.2. Study Population 

A total of 15005 individuals aged 3 years and over who were inhabitants of District 13 of Tehran which were under the coverage of three medical health centers, were selected using multistage cluster random sampling method. Medical health centers in this district benefited from a well-developed framework of experienced health volunteers (Rabet-e Behdasht) who played a critical role in the recruitment of people in the study and distributing some written educational documents among patients. Additionally they helped the study personnel to improve their knowledge and also urging the study population to participate in the intervention procedure. Age distribution and socioeconomic status of the population in district No. 13 is approximately representative of the overall population of Tehran (11).

A total of 5630 individuals aged 3 years and over under coverage of one of the three health centers with acceptable distance from two other centers, were considered for lifestyle modification using primary and secondary preventions for metabolic syndrome. For the present study all of those aged 20-74 years who had returned for follow up after 3.6 years from both intervention and control groups were included.

**Figure 1.**
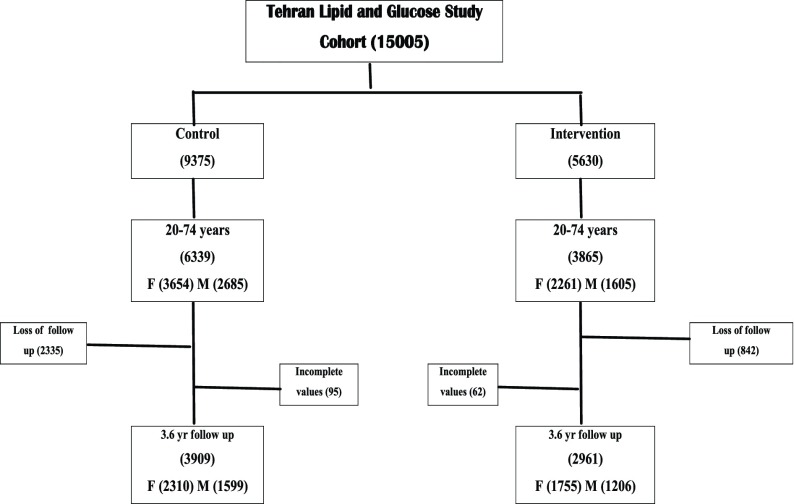
Flow Chart of Control and Intervention Groups. F=Female, M=Men, number in parenthesis denote number of individuals

From 9375 individuals in control group, 6339 were between 20-74 years of age and 4004 of them (63%) returned after 3.6 years. Those with missed values (n = 95) were excluded and finally data of 3909 subjects with complete values were used in this study. Of 5630 individuals in intervention group, 3869 were between 20-74 years of age and 3023 of them (54%) returned after 3.6 years. After excluding those with missed values again (n = 62), data of 2961 individuals with complete values were included as well (*Figure 1*). There was no difference in components of metabolic syndrome between responders and non-responders at baseline assessments.

### 3.3. Baseline Measurements

The following examinations were performed at the beginning of the study and after 3.6 years in both intervention and control groups. Trained social workers visited study participants 2-4 weeks before the examination and explained the process of study precisely. An appointment was made for the invited family and the date was confirmed through a phone call 2-5 days before examination, emphasizing the previous instructions including fasting of 12-14 hours before examination. On examination day the participants were interviewed for obtaining some demographic data or updating existing data. Then, the participants were referred to laboratory for blood sampling.

### 3.4. Medical History and Clinical Examinations

All invited participants to the TLGS unit, were interviewed by trained physicians to obtain past medical history and to complete a 110-item questionnaires regarding familial history of NCD, smoking habits, reproductive history and physical activity. Thereby brief physical examination was performed including anthropometric measurements. Moreover Trained dietitians collected dietary data in one tenth of the participated family. 

The participants remained seated for at least 15 minutes, then a qualified physician measured blood pressure two times using a standard mercury sphygmomanometer calibrated by Iranian Institute of Standards and Industrial Researches. The cuff was placed on the right arm, which was at the heart level and inflated in as high rate as possible until the cuff pressure was 30 mmHg above the level at which the radial pulse was disappeared. There was at least a 30-second interval between these two separate measurements, at last mean of two measurements was recorded as the participant’s blood pressure. The systolic blood pressure was defined as the hearing of the first sound (Korotkoff phase 1), and the diastolic blood pressure was defined as the disappearance of the sound (Korotkoff phase 5) during deflating the cuff at a rate of 2-3 mm per second. 

Anthropometrical measurements were taken with shoes removed and the participants wearing light clothing. Weight and height were measured according to the standard protocol (13). Waist circumference was measured at the level of the umbilicus. Body mass index (BMI) was calculated by dividing the weight in kilograms to the square of height in meters (14).

### 3.5. Assessment of Physical Activity

Lipid Research Clinic questionnaire was used for assessment of physical activity before intervention (15). On the basis of the standards of the Lipid Research Clinic, the participants were divided into 4 physical activity groups: highly active (people engaged in heavy sports activities who consider themselves more active than their partners); moderately active (people who participate in heavy sports activities and consider themselves as active as their partners); mildly active (people not involved in heavy sports but who consider themselves more active than their counterparts); and non active (people not engaged in heavy sports activity who don`t consider themselves as active as their partners). 

After assessing intervention physical activity using the modifiable activity questionnaire (MAQ) all respondents were asked if they had participated in any moderate exercise and vigorous exercise in their leisure time during recent one year prior to the interviews. We defined “leisure time physical activity” as performing three or more days of vigorous-intensity activity for at least 20 minutes, or five or more days of moderate-intensity activity or walking for at least 30 minutes, or five or more days of any combination of walking, moderate or vigorous-intensity activities achieving a minimum of at least 600 MET (metabolic equivalent task)-minutes per week (16). Those out of this definition were considered as physically inactive.

### 3.6. Assessment of Nutrition Status

Day to day variation of 24-hr dietary recalls was evaluated in 132 randomly selected subjects, comparing dietary data by repeated 24-hr dietary recalls every month (17).

### 3.7. Lifestyle Interventions

Interventions were aimed at lifestyle modification through primary and secondary preventions of metabolic syndrome by improving nutrition and dietary patterns, increasing physical activity levels, and smoking cessation. A total of 5630 people from District 13 were recruited for interventions. 

Based on knowledge, attitude and practice (KAP) study results which was conducted on a sample of 826 adults, dietary interventions and nutritional education protocols were designed. The KAP study included questions related to factors of weight changes, fat sources, fibers, snacks, food varieties including dairies, grains, fruits, vegetables, nuts, meats, sweets, pats, etc. The KAP study provided clues on and points to implement interventions on healthy nutritional practice and what is needed to be changed. In the beginning of intervention, nutrition education classes were held four days a week and in average 12 adults took part in each session where they were instructed using face to face consultation, showing films and slides along with nutrition recommendations; applicable guidelines for improving healthy dietary patterns were employed to instruct them. In addition, healthy nutrition messages were written in health newsletter called “well-being message” which were distributed every season. Pamphlets, brochures and booklets written on smoking, nutrition, physical activity and coping with stress were distributed 2-4 times a year to all families. In religious meetings lectures about NCD, their complications and prevention by modifying lifestyle strategies and acquiring healthy nutrition patterns were presented. Additional face to face interviews, group meetings and advisory clinics were arranged upon requests of individuals.

School-based lifestyle modification program was designed as a multidisciplinary health promotion program concentrating on a population approach which was underpinned by the health promoting schools philosophy. The program targeted at the whole school community including students, parents, teachers, staff, and the school environment. It was intended to alloting anti-tobacco effects, encourage healthy nutrition consumption and increase physical activity practices and not simply distributing some knowledge in schoolchildren. Classroom teaching, formation of school “health team” and peer-teaching approach were also implemented.

Secondary prevention in nutrition intervention was implemented in nutrition clinics and for subjects with diseases such as diabetes, overweight and obesity, dyslipidemia and hypertension who did not have family physicians and/or volunteered to attend these clinics.

### 3.8. Definitions

According to the NCEP-ATPIII criteria, metabolic syndrome was defined as the presence of three or more of the following risk factors: abdominal obesity (waist circumference > 102 cm in men or > 88 cm in women); triglycerides ≥ 1.7 mmol/L; HDL cholesterol < 1.03 mmol/L in men or < 1.29 mmol/L in women; blood pressure systolic ≥ 130 or diastolic≥ 85 mmHg; and (fasting plasma glucose) FPG ≥ 5.6 mmol/L (1).

### 3.9. Biochemical Measurements

All measurements were performed on serum samples obtained after 12-14 hours of fasting. Serum triglyceride was measured by kits from Pars Azmon Inc., Iran, using enzymatic calorimetric tests with glycerol phosphate oxidase, HDL-C was measured after precipitation of the apolipoprotein B containing lipoproteins with phosphotungistic acid. Assay performance was monitored in every 20 tests interval using the lipid control serum, Precinorm (normal range) and Precipath (pathologic range) were used wherever applicable (Boehringer Mannheim, Germany; cat. no. 1446070 for Precinorm and 171778 for Precipath). Lipid standard (C.f.a.s, Boehringer Mannheim, Germany; cat. no. 759350) was used to calibrate the Selectra 2 auto-analyzer (Vital Scientific, Spankeren, Netherlands) on all days of laboratory analyses. Serum glucose concentrations were assayed using enzymatic colorimetric method with glucose oxidase technique. Assay performance was monitored at every 20 tests intervals using the glucose control serum, Precinorm (normal range) and Precipath (pathologic range) wherever applicable (Boehringer Mannheim, Germany; cat. no. 1446070 for Precinorm and 171778 for Precipath). Glucose standard (C.f.a.s, Roche, Germany; cat. no. 759350) was used to calibrate the Selectra 2 auto-analyzer on all days of laboratory analyses. All samples were analyzed when internal quality control met the acceptable gold standard. Inter- and intra-assay coefficients of variations were both 2.2% for serum glucose and .6% and 0.6% for serum triglyceride, respectively, in both phases of study. Fasting serum insulin was measured with ELISA insulin Accbind kit, Monobin Inc. Costa Mesas Co. USA. Homostatic model assessment (HOMA-IR) was calculated with the following formula (18).

### 3.10. Statistical Analysis

Statistical analyses were performed using the Statistical Software package version 15.0 (SPSS Inc., Chicago, IL). All continuous data with normal distribution are expressed as mean ± SD. Triglycerides levels were log transformed. Continuous and dichotomized variables were compared at baseline and after 3.6 years, using the paired T and the McNemar tests, respectively. In each phase of the study, correlations between metabolic syndrome and its components in two groups were determined with logistic regression adjusted for age, sex and medications for dyslipidemia, hypertension and hyperglycemia. In the second phase (after intervention) adjustment was also made for the prevalence of each variable in the first phase (before intervention). To obtain the *P* value for change, differences in the prevalence of metabolic syndrome and its components in phase 1 and 2 were calculated in each group using mean weight and the significance changes were assessed with Z test. Student T test was used to obtain *P* value for changes that had occurred before and after intervention for each variable. We employed analysis of covariance for correlation among means of variables adjusted for age, sex, weight at entry, level of education, cigarette smoking and medications consumption for dyslipidemia, hypertension and hyperglycemia; in addition in phase II, adjustment for mean of variable before intervention was also performed. For dietary data the within-person coefficient of variation (CV) obtained from the analysis of variance on repeated days of dietary intake; the square root of the within-person variance is the within-person standard deviation, and this value divided by the mean is the within-person CV (11). Probability values < 0.05 were considered as statistically significant.

## 4. Results

Altogether 6870 subjects 4065 women and 2805 men aged 20-74 years attended in both phase of the study. They were 1755 women and 1206 men in intervention and 2310 women and 1599 men in control groups. Mean age was 43.3 ± 14.0, and 43.8 ± 14.1 years in control and intervention groups, respectively. There was no significant difference in age, sex, literacy, general and abdominal obesity, serum concentrations of lipids, glucose and insulin, HOMA-IR, physical activity, and other baseline variables between intervention and control groups (*Table 1*).

**Table 1.** Baseline Characteristics of the Intervention and Control Groups

Characteristic	Control (n=3909)	Intervention (n=2961)	
**Male, %**	40.9	40.7	
**Literacy, %**	90.4	90.6	
**Age, year**	43.3 ± 13.9 ^a^	43.8 ± 14.1	
**Marital status (% married)**	83.1	82.5	
**Daily cigarette smoker,%**	11	9	
**Body mass index, kg/m** **^2^**	27.1 ± 4.6	27.1 ± 4.6	
**Waist circumference, cm**	88.7 ± 12.1	88.9 ± 12.1	
**Waist to hip ratio**	0.87 ± 0.08	0.87 ± 0.08	
**Systolic blood pressure, mm/Hg**	120 ± 19	120 ± 19	
**Diastolic blood pressure, mm/Hg**	78 ± 10	78 ± 11	
**Fasting serum glucose, mg/** **dL**	98.9 ± 34.2	99.3 ± 35.3	
**Serum insulin, mIU/L ** **^b^[Table-fn fn3101]**	7.52 ± 5.64	7.08 ± 5.77	
**HOMA-IR**	5.5 ± 4.9	5.2 ± 4.8	
**Serum cholesterol, mg/** **dL**	212 ± 47	212 ± 46	
**Serum triglyceride, mg/** **dL**	174 ± 121	174 ± 120	
**Serum LDL-C, mg/** **dL**	135 ± 38	136 ± 38	
**Serum HDL-C, mg/** **dL**	42.3 ± 10.9	42.3 ± 11.0	
**Physical activity, %**			
Highly active	26.5	23.4	
Moderately active	18.2	16.2	
Mildly active	30.5	34.4	
Not active	24.8	26.0	

^a^ Except for percentage (%), all numbers are mean ± SD

^b^ Serum insulin concentration, and HOMA-IR were determined in randomly selected 260 and 83 persons of control and intervention groups, respectively.

There was no significant difference in the prevalence of metabolic syndrome and its components at the baseline between 2 groups (*Table 2*). After 3.6 years, the prevalence of metabolic syndrome increased from 37.7% to 41.2% (*P *< 0.001) in intervention group, and from 38.6% to 44.7% (*P *<0.001) in control group. The rise in the prevalence of metabolic syndrome was more prominent in control as compared to intervention group (*P *< 0.002 for the change between groups). The age and sex adjusted odds ratio (OR) in the intervention group compared with the control group was 0.84 (95% CI, 0.75-0.95).

**Table 2.** Prevalence of Metabolic Syndrome and Its Components in the Intervention Group (n = 2961) and Control Group (n=3909) at Baseline and After 3.6 years: Tehran Lipid and Glucose Study

	Baseline			After 3.6 Years					P value for Change
	Int, % ^b,f^	Cont^f^	*P* value Between Groups^c^	Int^a^	*P* value From Baseline to Year 3.6	Cont	*P* value From Baseline to Year 3.6	*P* value Between Groups^c^	
**Metabolic syndrome**	37.7	38.6	0.39	41.2	< 0.001	44.7	< 0.001	< 0.002	< 0.001
**Abdominal obesity** **^a^**	37.1	36.0	0.50	46.6	< 0.001	48.3	< 0.001	< 0.014	< 0.001
**Elevated fasting glucose ** **^a^**	24.6	24.3	0.89	23.7	0.28	29.0	<0.001	< 0.001	< 0.001
**Elevated triglyceride** **^a^**	49.8	50.3	0.58^d^	44.7	< 0.001	47.8	< 0.002	0.014^d^	<0.001
**Low HDL cholesterol ** **^a^**	69.2	69.9	0.84^d^	76.1	< 0.001	82.2	< 0.001	< 0.001^d^	< 0.001
**Elevated blood pressure** **^a^**	34.6	34.2	0.94^e^	31.1	< 0.001	29.5	<0.001	0.25^e^	< 0.014

^a^Abdominal obesity: waist circumference ≥ 102 cm in men and ≥ 88 cm in women. Elevated fasting glucose: fasting plasma glucose ≥ 5.6 mmol/L. Low HDL cholesterol: HDL cholesterol < 40 mg/dL (< 1.03 mmol) in men and < 50 mg/dL (1.3 mmol/L) in women. Elevated triglycerides: serum fasting triglycerides ≥150 mg/dL (≥1.7 mmol/L). Elevated blood pressure ≥ 130 mmHg, diastolic blood pressure ≥ 85 mmHg, and/or use of antihypertensive medication

^b^Adjusted for age and sex

^c^Adjusted for age, sex, and baseline value

^d^Adjusted for lipid medications.

^e^Adjusted for blood pressure medications

^f^Abbreviations: Int, Intervention; Cont; Control

The change of different components of metabolic syndrome in intervention and control groups at baseline and after 3.6 years is shown in *Table 2*. After 3.6 years, decrease in the prevalence of elevated blood pressure and elevated triglycerides levels occurred in both groups compared with baseline values. The prevalence of elevated fasting glucose increased in control group, but not in intervention group. The prevalence of abdominal obesity and low HDL cholesterol increased significantly in both groups after 3.6 years, as compared to baseline values.

However, comparison between groups showed that the prevalence of metabolic syndrome and 4 of its 5 components, (abdominal obesity, elevated fasting glucose, elevated triglyceride and low HDL cholesterol) were more prominent in the control group, as compared to intervention group. Comparisons showed that lifestyle intervention reduced abdominal obesity (OR 1.24, CI 1.07-1.44), elevated fasting glucose (OR 1.67, CI 1.43-1.95), elevated triglyceride (OR 1.18, CI 1.04-1.34) and low HDL cholesterol (OR 1.52, CI 1.32-1.76), adjusted for age, sex and baseline values. Changes in the prevalence of metabolic syndrome in various age groups are shown in Table 3. Although the prevalence increased in all age groups in both intervention and control subjects, the increase was less in intervention group as compared to control group in all age groups, except for subjects of 20-29 years old.

*Table 4* compares changes in various parameters of metabolic syndrome in various age groups. Significantly fewer rises in percentages of abnormality in components of metabolic syndrome was obvious in all age groups in intervention subjects. Change between intervention and control groups was more evident in the frequency of elevated fasting glucose and low HDL cholesterol after 3.6 years of intervention. Percentage of elevated fasting glucose increased in all age groups in control subjects, but not in the intervention individuals, with significant decrease in the percentage of hyperglycemia in the elderly subjects.

**Table 3.** Prevalence of Metabolic Syndrome in Various Age Groups of the Intervention (n = 2961) and Control Groups (n = 3909) at Baseline and After 3.6 Years: Tehran Lipid and Glucose Study

Age Group, y	Baseline			After 3.6 Years					P value for Change
	**Int, % ^a^**	**Cont ^c^**	***P* value Between Groups ^b^**	**Int**	***P* value From Baseline to Year 3.6**	**Cont**	***P* value From Baseline to Year 3.6**	***P* value Between Groups ^b^**	
**20-29**	10.1	11.8	0.33	13.2	0.096	13.9	0.165	0.86	0.158
**30-39**	24.7	22.4	0.87	28.5	0.08	32.5	< 0.001	0.06	< 0.001
**40-49**	44.1	46.1	0.68	48.4	0.053	3.6	< 0.001	0.08	< 0.014
**50-59**	57.8	57.1	0. 90	62.0	0.089	63.5	< 0.001	0.46	< 0.069
**60-69**	57.3	60.3	0.54	58.8	0.672	64.8	< 0.047	0.12	< 0.009
**≥ 70**	45.6	51.5	0.98	47.4	1.00	63.3	< 0.035	0.13	< 0.001

^a^Adjusted for age and sex.

^b^Adjusted for age, sex, and baseline value.

^c^Abbreviations: Int, Intervention; cont, Control

**Table 4.** Comparison of Changes in Parameters of Metabolic Syndrome in Various Age Groups of Intervention and Control Subjects After 3.6 Years of Follow up: Tehran Lipid and Glucose Study

Age Group, y	Change Between Intervention and Control Groups^a^,% (*P* value)				
	Abdominal Obesity^b^	Hypertension^c^	Elevated Fasting Glucose	Elevated Triglycerides^d^	Low HDL Cholesterol^d^
**20-29**	-0.7 (0.357)	+1.5 (0.071)	-3.5 (< 0.001)	-4.4 (< 0.003)	-3.9 (< 0.029)
**30-39**	-5.3 (< 0.003)	-1.6 (0.023)	-4.6 (< 0.001)	-3.95 (< 0.001)	-4.5 (< 0.016)
**40-49**	-1.4 (0.245)	-3.2 (0.256)	-6.8 (< 0.011)	-3.2 (< 0.003)	-6.7 (< 0.001)
**50-59**	-0.1 (0.48)	+0.8 (0.441)	-10.9 (< 0.001)	-0.6 (0.359)	-11.1 (< 0.001)
**60-69**	-7.0 (< 0.001)	+7.4 (< 0.001)	-3.2 (< 0.001)	+0.6 (0.39)	-3.2 (< 0.074)
**70-74**	+1.8 (0.371)	-10.6 (< 0.001)	+1.8 (< 0.001)	-15.03 (< 0.001)	+1.8 (< 0.383)

Dietary data did not show significant difference in energy intake and macronutrient consumption between two groups at baseline and after intervention. The within person CV was large at 32%, ranging from 30% in women to 42% in men.

Most individuals in control and intervention groups were mildly active and non active before intervention, 83% and 84%, respectively. The corresponding values were 78% and 72% (*P* = NS) after intervention. Chance for being less active was significantly higher in control men as compared to intervention men after 3.6 years: OR = 1.2 (1.01-1.44, *P* < 0.05).

Regular monitoring of drug intake for diabetes, dyslipidemia and hypertension showed that both before and after lifestyle interventions no significant difference in drug intake was seen between two groups.

## 5. Discussion

In this community based intervention study we found that a 3.6 year of lifestyle intervention slowed the rise in the prevalence of metabolic syndrome and some of its components. regarding our knowledge this is the first large population based study that shows the effectiveness of lifestyle modifications in the reduction of rise in metabolic syndrome prevalence in a community.

Few studies have been conducted on lifestyle modification for reducing prevalence of metabolic syndrome. Weight loss was the most important underlying preventive mechanisms in order to lessen metabolic syndrome prevalence (5-8). Weight reduction of > 10% of initial body weight leads to greater fall in the prevalence of components of metabolic syndrome, as compared with weight loss of < 10% which induces less efficacy (18). It has been shown that the adoption of various dietary patterns may normalize the components of the metabolic syndrome (19-21), including lowering blood pressure in hypertensive patients with metabolic syndrome (22). In the present study, change in the prevalence of metabolic syndrome and 4 of its 5 components were significantly lower in intervention group as compared to control group after a 3.6 year of intervention. Although the prevalence of 2 of 5 components (abdominal obesity and low HDL cholesterol) increased in the intervention group, despite the fact, these increments were significantly smaller than those in control group. The prevalence of fasting hyperglycemia was unchanged in intervention group, after 3.6 years while the prevalence of this component increased significantly in the control group. Fall in the prevalence of elevated triglyceride was also more prominent in intervention group, as compared to control group.

Abdominal obesity and insulin resistance are the main elements of metabolic syndrome (20, 22). A change in body weight strongly correlates with an augmentation in insulin sensitivity (23). While abdominal obesity increased in both groups after 3.6 years, but the increment in intervention group was lower than that in control group. It may proposed that this difference may also contribute to higher later cardiovascular diseases risks, as abdominal obesity which correlates well with this unwanted events (24).

Lack of change in the prevalence of increased fasting glucose in the intervention group compared with its increase in the control group is significantly of importance. Recent studies have shown that lifestyle intervention reduces the risk of progression from impaired glucose tolerance to overt type 2 diabetes (6). The findings of present study is in concordance with the above findings that without intervention the prevalence of fasting hyperglycemia increases, but popularization of this concept to a community scale, shows that lifestyle intervention in general population could also reduce the risk of rise in fasting glucose.

Rise in the percentages of abnormalities in many components of metabolic syndrome was less prominent in both gender and all age groups. This important finding points to the parallel effectiveness of lifestyle interventions in all members of the study population.

All recommendations for clinical management of the metabolic syndrome can be summarized in lifestyle changes, healthy diets and more exercises, at first-line interventions and consequently drug therapy when necessary, along with strategies for cardiovascular diseases prevention (7, 25, 26). The ATP III, in its update, states that “in those with the metabolic syndrome, individualized counseling for weight loss, regular exercise, and therapeutic diet may be particularly effective in helping to improve plasma triglyceride and HDL cholesterol values while offering promise to reducing the burden of medication required to improve the risk factor profile” (27). However, most current health providing systems in various countries do not provide tools or supports to properly initiate lifestyle changes; for example, workplaces, social and cultural systems do not provide opportunities or facilities for increasing physical activities compliance among people. 

It is well documented that physical activity has several beneficial effects on most components of metabolic syndrome including elevated blood pressure, central obesity and insulin resistance (22, 23, 28). This may due to a direct effect or an effect resulted from promoting weight reduction (29, 30). Changes in physical activity have been added to healthcare instructions in most therapeutic interventions for preventing promotion of metabolic syndrome (22, 29). However, it is believed that dietary changes are considered as the core strategy of the management, with weight reduction playing as a key role and moreover exercise having an additional favorable effect. In the present study, small difference in physical activity was only seen in men after intervention; however, Lipid Research Clinic questionnaire which was employed in this study is a crude tool for the assessment, while other quantities instruments might have shown more changes.

Attention to behavioral factors including cognitive readiness and psychological parameters that might influence individual’s ability to reach lifestyle ideals helps people to adhere to multifaceted and demanding lifestyle modifications. Context of behavioral intervention may include goal setting, self-monitoring, problem-solving, Control of stress stimulus, management of high-risk situations and relapse prevention (31).

In a recent review, Fappa et al. evaluated the effectiveness of lifestyle intervention by critically exploring patients adherence to the recommendations, in 6 randomized and one non-randomized controlled trials and 5 without control group intervention trials, they found that greater adherence was correlated with greater improvements in the components of metabolic syndrome (32). However, the evidence was limited regarding respective approach to specific behavioral or motivational factors in helping patients to adhere to the multifaceted lifestyle changes.

The present study has a few limitations. Nutritional data before and after intervention collected only in a few individuals and did not show significant changes after intervention. It is north worthy that total calorie and nutrients intake in free-living persons differs from day to day and seasonally (33, 34). The within-person CV in our population proved to be large that tends to be similar in studies in India and China (35). Measurements of dietary intake based on small numbers of 24-h DRs per subject may provide a reasonable mean estimate for a group, but the standard deviation will be greatly over-estimated (33). Such information directly influences the detection of differences between treatment and control groups in experimental or interventional studies. We used Lipid Research Clinic questionnaire for baseline measurements of physical activity; however, the results were not conclusive and the questionnaire was considered not to be appropriate for TLGS population (12). The strength of this study is a well controlled intervention at the community level with a large number of participants who completed 3.6 years of follow up. The novelty of the work resides in demonstrating the effectiveness and efficiency of the style of intervention used in terms of real life in the community.

In conclusion, comprehensive life style modification could prevent the rise in the prevalence of metabolic syndrome at the community level. The intervention is effective in reducing or halting abnormalities in all components of metabolic syndrome and particularly in elevated fasting glucose and low HDL cholesterol. It is of outmost interest to study outcomes of cardiovascular and other non-communicable diseases after longtime community life style modification.
